# Dentin Erosion: Method Validation and Efficacy of Fluoride Protection

**DOI:** 10.3390/dj5040027

**Published:** 2017-10-06

**Authors:** Clifton M. Carey, William Brown

**Affiliations:** School of Dental Medicine, University of Colorado, 12800 E. 19th Ave., MS8310, Aurora, CO 80045, USA; WILLIAM.J.BROWN@UCDENVER.EDU

**Keywords:** dentin, erosion, fluoride, citric acid, prevention, tooth

## Abstract

The aging population experiences more gingival recession and root exposure which increases the opportunity for dentin erosion. This study tested the use of transverse microradiography (TMR) methods to assess dentin erosion and the interaction between fluoride and citric acid on the amount of erosion in the dentin samples. In a 4 × 3 interaction experimental design, four fluoride concentrations (0.00, 25.0, 50.0, and 100.0 mg/L) and three citric acid concentrations (0.0, 0.25, and 1.00%) were combined to form 12 experimental solutions. Forty-eight dentin samples were placed in the experimental solutions for 1 and 4 h and the amount of surface lost was determined by TMR methods. The resolution of the TMR method was 0.9 μm per pixel with a 0.1% and a 5% confidence interval of ±4.2 μm. Dentin erosion increased with the concentration of citric acid and time, the erosion decreased when concentration of fluoride was increased. Effects due to fluoride and citric acid concentrations individually, and their interaction on the amount of erosion observed was statistically significant (*p* < 0.0001). This study found that TMR methods are appropriate and that 25.0 mg/L was the optimal fluoride concentration to protect dentin from a 1.00% citric acid challenge.

## 1. Introduction

Dental erosion is defined as the irreversible loss of tooth surface due to acids not generated by microbiological sources [1,2] and is not tooth softening, subsurface demineralization, or caries [3]. Specifically, dental erosion is caused by acids in food, beverages, medications, recreational or occupational acidic exposures [4], and from reflux of stomach acids [5]. The erosion can be aggravated by slow acid clearance due to low salivary flow [6]. Dental erosion is distinguished from other forms of tooth surface loss such as erosive tooth wear, or abrasion in that the loss of tooth surface is solely due to exposure of the tooth to acids. Worldwide, there is an increase in the consumption of acidic beverages that has led to a high prevalence of dental erosion [7,8,9,10]. 

Enamel erosion has been reported to be independent of the age of a patient [11], however dentin erosion is likely to be age dependent due to increased amounts of gingival recession, root exposures, and accumulated risk factors. As increasing numbers of people are retaining their teeth throughout their lifetimes, gingival recession has become a more prominent issue. Gingival recession exposes root dentin to the oral environment which increases the opportunity for acidic erosive challenges to the dentin. This exposed dentin can then be eroded, leading to the need for restoration or eventually tooth loss. The prevention of erosion by topical fluoride pretreatment seems to be only partially effective and requires an intensive fluoridation regime to achieve significant protection [12]. Diamanti et al. [13] reported that pretreatment with sodium fluoride concentrations of 1450 ppm F to 5000 ppm F were effective in reducing erosion. Similarly, Moron et al. [14] reported that dentin erosive wear is significantly reduced by high fluoride containing dentifrices. 

While prevention of either enamel or dentin erosion seems to require intensive topical fluoride therapy [15], there is little in the literature about the preventive capacity of fluoride when it is present in the acid solution. We have observed that enamel erosion can be halted when relatively little added fluoride is present in the acidic challenge [16]. The purpose of this study was to assess the effect of fluoride concentrations in the acid challenge for the preservation of dentin. The interaction between fluoride concentration (F) in the acid and the concentration of citric acid (CA) on the amount of erosion in the dentin samples was also studied. 

There are a number of methods for determining dentin erosion. These vary from measuring the loss of mass [17], to confocal laser scanning microscopy, profilometry, Knoop Hardness, and transversal microradiography [18]. Schwendicke et al. [18] compared these later four methods and found that transverse microradiography (TMR) and confocal laser scanning microscopy are reliable methods. It is clear that these two methods have sensitivity on the micron scale, and are thus capable of the measurement of small amounts of surface loss. TMR is considered the gold standard for determining changes in mineral density and the assessment of enamel erosive losses [19]. This study included the development of TMR techniques to determine erosive surface loss for dentin.

## 2. Materials and Methods

Our goal was to assess the relationship between citric acid (CA) concentration and the amount of fluoride (F) required to stabilize dentin surfaces during 1-h and 4-h erosive attacks.

- Evaluate the effect of fluoride concentration on dentin erosion
- Evaluate the effect of citric acid concentrations on dentin erosion
- Determine if there is an interaction between fluoride and citric acid

### 2.1. Experimental Design

We used a 4 × 3 factorial design (4 F concentrations × 3 CA concentrations) at two different lengths of exposure time to assess the impact of F, CA, and their interaction on dentin erosion. Erosion was determined via transverse X-ray microradiography (TMR).

The Colorado Multiple Institutional Review Board determined that the use of human teeth extracted for therapeutic reasons which were collected from the waste stream constitutes non-human research. Human teeth were collected from the waste stream of the School of Dental Medicine and stored in a sterilizing tooth storage solution. More than 50 adult teeth with caries-free roots were selected for these experiments.

### 2.2. Sample Preparation

**Figure 1:**
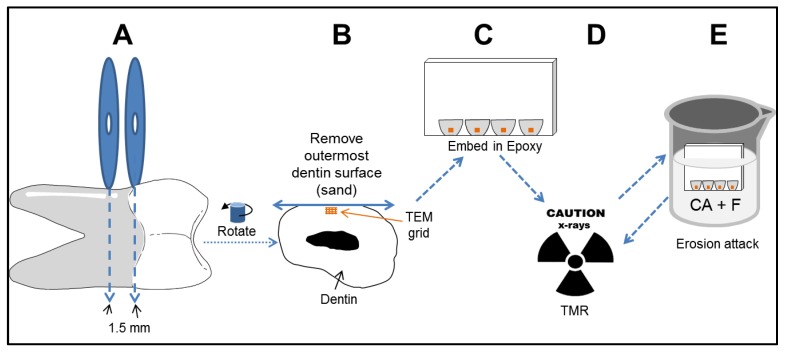
Schematic for sample preparation and erosion challenges.

Non carious roots were removed by slow speed water cooled diamond saw just below the dentin-enamel junction as shown in Figure 1a. The roots were sliced into 1.5 mm thick samples and a piece of TEM grid was attached to the cut surface of the dentin near the surface with a small amount of poly(methyl methacrylate) glue, see Figure 1b. The sample was then embedded in X-ray transparent epoxy (1c). This section of root typically has a low fluoride concentration within the first 20 μm from the surface [20,21]. However, the outermost surface of 50 to 70 μm was removed by abrasion on wet 1200-grit sandpaper to assure exposure of fresh dentin (1b). No other surface preparation or sample selection was employed. Up to four samples were embedded into one epoxy sample holder (1c). Care was taken to prevent the samples from drying as the samples were prepared. Additionally the samples were suspended in water for 48 h to assure complete hydration. Forty-eight samples were prepared and randomly assigned to 12 groups of 4 samples each. The groups were 0% CA (0, 25, 50, 100) μg/mL F at pH 7.1; 0.25% CA (0, 25, 50, 100) μg/mL F at pH 3.68; and 1.0% CA (0, 25, 50, 100) μg/mL F at pH 3.60. The CA concentrations are those suggested by an international standard that screens oral rinses for erosive capacity [22].

### 2.3. Sample Erosion and Digital Imaging

Initial X-ray images of all samples were made following the methods described in Schmuck and Carey [19]. The exposure was 6 min at 80 kW at 20 cm distance from the X-ray source. The samples were placed directly on the emulsion side of holographic film (Intergraf Holography Supplies & Services, Kirkland, WA, USA) for the exposure along with an aluminum step wedge. The films were developed following manufacturer instructions and air dried. Digital TMR images were made of the X-ray images at 40× with a Meiji binocular microscope fitted with a high resolution digital camera.

Groups of four samples were exposed to 25 mL of three different CA concentrations at 0 (DI-H_2_O), 0.25% and 1.0% CA solutions that contained 0, 25, 50 and 100 μg/L F (ppm F) for 1-h. Citric acid solutions with concentrations of 0.25% (pH = 3.68 ± 0.05) and 1.00% (pH = 3.60 ± 0.05) were prepared using citric acid (Carl Roth, Karlsruhe, Germany) and sodium citrate (Sigma, St. Louis, MO, USA). These are the same CA erosion standards to be used for assessing erosion described in international standards [22]. These standards are designed for validating methods to assess hard tissue erosion. The samples were removed from the CA at 1 h and rinsed with DI-H_2_O prior to imaging. Digital TMR images were made for the 1-h erosion exposure and the samples placed in fresh CA/F solutions for an additional 3 h. The samples were rinsed with DI-H_2_O and final digital TMR images were made for the 4-h erosion.

### 2.4. Digital Analysis

**Figure 2:**
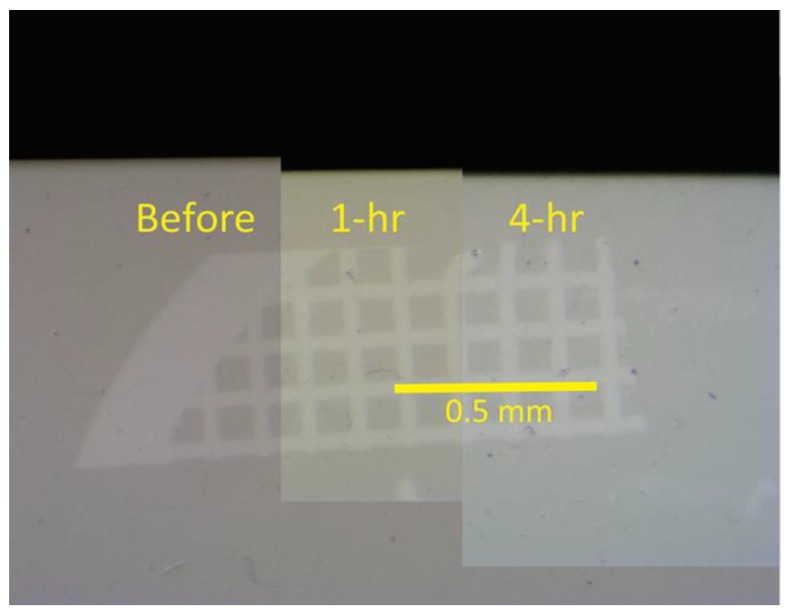
X-ray images aligned to show effect of time on citric acid erosion of dentin. Dentin sample X-rays taken before, and at 1 h and 4 h were aligned using the fiduciary TEM grid marker to assess surface mineral loss.

By use of Image-J software (NIH) the digital TMR images (time 0, 1, and 4 h) were converted to 32-bit grey scale images, and a rectangle was selected from the outside of the dentin sample through the TEM grid. The same rectangle was used for the time 0, 1, and 4 h images of each sample of dentin. The grey scale value of the pixels from the selected area was measured and placed on a spreadsheet for graphical analyses. The pixel density (grey scale) was adjusted for 0% mineral density (outside of the dentin sample) and 100% dentin density (within the dentin sample) for each sample image. This relative density data was then plotted for analysis of the amount of erosion loss for each sample. The scale of the image was calibrated with a digital image of a U. S. National Institute of Standards and Technology (NIST)-traceable millimeter scale at 1000 μm taken at the same magnification as the digital TMR images were made. The relative mineral density profiles of the three X-ray images of each dentin sample were aligned to the X-ray dense TEM grid revealing any change in the surface of the samples, see Figure 2. The initial profile distance (time 0) minus the 1-h profile distance at 20% mineral density is the amount of surface mineral lost to erosion attack for 1 h. Likewise, the difference between initial profile at time 0 and the 4-h profile at 20% mineral density is the amount of surface mineral lost due to 4 h of erosive attack. Twenty percent density was chosen because it is above the density of background materials such as protein or water and is clearly tooth structure. Positive numbers represent the μm of dentin lost due to erosion, negative numbers indicate that the surface appeared to increase.

### 2.5. Statistical Evaluations

The accuracy and reproducibility of the TMR measurement method was determined from 15 repeated measures of a NIST traceable millimeter scale at 1000 μm at the same magnification as the samples were imaged. The erosion loss data for the 1-h and 4-h experiments were tabulated, graphed, and evaluated by two-way ANOVA at each time interval with CA concentration and F concentration as the independent variables. The differences between the 12 groups of the 1-h and 4-h experiments were evaluated via post hoc ranked comparison (Student–Newman–Keuls).

## 3. Results

The limit of detection for these experiments was 0.9 μm per pixel of the digitized X-ray image. The resolution of the TMR method was 0.9 μm per pixel with an accuracy determined by repeated measurement (*n* = 15) of a 1000 μm standard at the same magnification as the samples (40×) was 1001.2 μm (0.1%) with a confidence interval of ±4.2 μm at α = 0.05. The power of these experiments to statistically detect a difference of ±15 μm surface loss with an observed pooled standard deviation of 7.3 μm at an α = 0.05 with *n* = 4 per group was 0.97.

**Figure 3:**
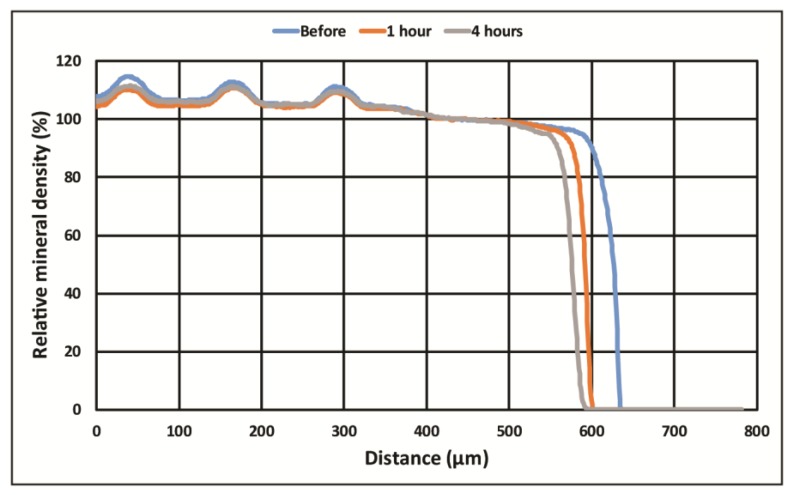
An example of a dentin sample exposed to 0.25% CA/0-F solution showing the aligned dentin profiles from which the surface loss is determined. The blue line is the initial relative mineral density profile at time 0; the orange line is the relative mineral density at 1 h; and the grey line is the relative mineral density at 4 h. The mineral density profiles of the three X-ray images of the dentin are aligned to the X-ray dense TEM grid, which is the three peaks located at 50, 160, and 270 μm from the left edge of the image. The difference between the blue line (initial profile) and the orange line (1 h) at 20% mineral density is the amount of surface mineral lost. Likewise, the difference between the blue line and the grey line (4 h) at 20% mineral density is the amount of surface mineral lost. The erosive loss for this sample was 35.2 μm in 1 h and 48.8 μm in 4 h.

**Figure 4:**
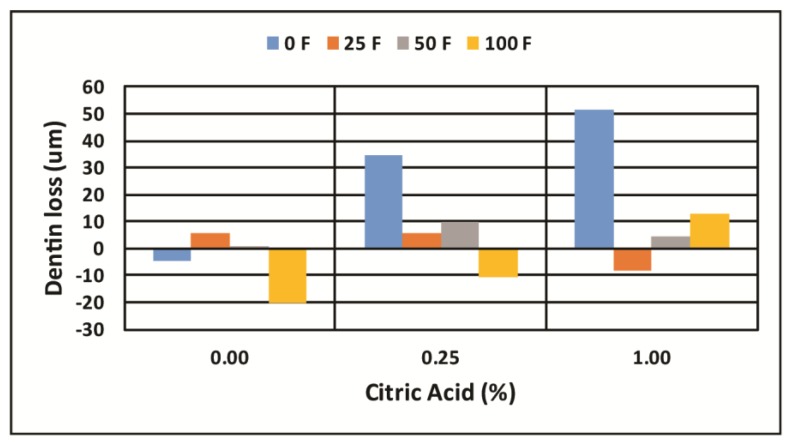
The amount of surface lost at 1 h of erosive challenge in the 12 experimental CA/F solutions. Negative values indicate surface gain, positive values indicate erosive loss of the dentin surface.

**Figure 5:**
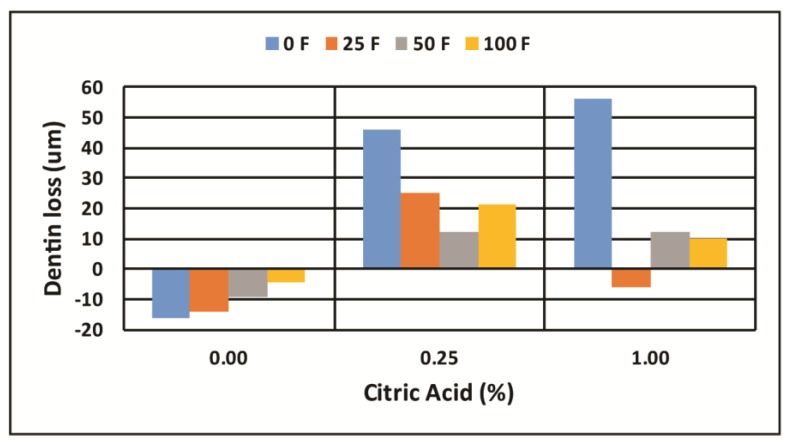
The amount of surface lost at 4 h of erosive challenge in the 12 experimental CA/F solutions. Negative values indicate surface gain, positive values indicate erosive loss of the dentin surface.

Figure 2 shows the X-ray images taken before and at 1 h and 4 h of erosion of a dentin sample aligned using the fiduciary marker to assess surface mineral loss. Figure 3 shows the aligned relative mineral density graphs at each time from which the surface loss is determined. The mean and standard deviation for each experimental group are shown in Table 1 for the 1-h experiments and Table 2 for the 4-h experiments. A positive mean value indicates the erosion loss, a negative mean value indicates a mineral gain on the surface. The letters associated with each experimental group (A, B, C, etc.) designate experimental groups that are statistically different at *p* < 0.05 (groups with the same letter are not statistically different) as determined by two-way ANOVA followed by post hoc ranked comparisons. Experimental groups with combinations of letters are members of each group at *p* > 0.05. Figure 4 and Figure 5 are the charts for visualization of these data.

The two-way ANOVA (with replication) found significant differences due to the concentration of CA (*p* < 0.001), the concentration of F (*p* < 0.001) and a significant interaction between citric acid (CA) and fluoride (F) at *p* < 0.001 for both the 1-h and 4-h experiments (Table 3).

## 4. Discussion

The methods used to determine dentin erosion are taken from Schmuck and Carey [19] which describe methods to determine enamel erosion. The high sensitivity at 0.9 μm and low variations observed in these experiments allow for sensitive detection of small amounts of dentin surface loss due to erosion. Our results are consistent with findings reported by Schwendicke et al. [18] where TMR methods are sufficiently sensitive and reproducible for use in dentin erosion experiments.

At 0 added F the strength of the CA was proportional to the amount of erosion observed for both the 1-h and 4-h experiments. At 0% CA where the samples were exposed to F solutions at neutral pH, we observed an apparent increase in surface relative to the starting surface. This was especially prominent at 4 h. The increase of mineral onto the surface could be due to the adsorption of F to the surface protein resulting in an X-ray dense accretion on the surface of the samples. Normally, the protein layer at the surface of the dentin would not be sufficiently dense to be observed at 20% relative mineral density.

The erosive losses at 4 h were much less when F was present than when F was not present in the CA. This shows that when F is present at the site of crystal dissolution it has the effect of interfering with that dissolution process and further tends to stabilize the mineral crystal structure against acid attack. When F was present in the CA solutions a significant reduction in erosion was observed at the 25 ppm F, and that significant reduction was maintained at 50 and 100 ppm F in the CA solutions. The effect of 25 ppm F was greater when present in the stronger 1.0% CA than when in the 0.25% CA at both the 1-h and 4-h erosion challenges as seen in Figure 1 and Figure 2. This result may be because the 25 ppm F concentration is sufficient to be present at all of the CA attack sites on the dentin surface, where higher F concentrations could be forming calcium precipitates thus lowering the activity of F ions in solution. The results of these dentin erosion experiments closely follow the results that have been reported in similar experiments on enamel erosion that found that moderate amounts of fluoride exposure during the erosive challenge can stop enamel erosion [16].

## 5. Conclusions

Based on these data and the methods used, 25 mg/L (ppm) fluoride is the optimal amount of fluoride to prevent dentin erosion. When fluoride is present at the time of the erosive challenge, it will prevent erosion. Clinically, low amounts of F added to highly acidic beverages may have a large effect in reducing dentin erosion. Should the results of this in vitro research be validated in vivo then the presence of relatively small amounts of fluoride at the dentin surface from beverages would be particularly useful for older populations who are experiencing a greater frequency of exposed root surfaces which would be at risk of erosion.

## Figures and Tables

**Table 1:** Dentin surface lost due to erosion (μm) for each of the 12 experimental groups at 1 h of erosive exposure. Values are average ± standard deviation, *n* = 4 for each group. A positive mean value indicates the erosion loss, a negative mean value indicates a mineral gain on the surface. The letters associated with each experimental group (A, B, C, etc.) designate experimental groups that are statistically different at *p* < 0.05 (groups with the same letter are not statistically different) as determined by two-way ANOVA followed by post hoc ranked comparisons. Experimental groups with combinations of letters are members of each group at *p* > 0.05.

1 h	Fluoride (ug/g)											
Citric Acid (%)	0			25			50			100		
**0.00**	−4.9	±	3.3 BCD	5.5	±	3.8 B	0.4	±	3.6 BCDE	−20.5	±	5.3 A
**0.25**	34.7	±	5.2 G	5.6	±	4.5 DEF	9.8	±	4.8 EF	−10.4	±	5.8 B
**1.00**	51.7	±	15.2 G	−8.1	±	7.3 BC	4.4	±	8.9 CDEF	13.1	±	4.1 F

**Table 2:** Dentin surface lost due to erosion (μm) for each of the 12 experimental groups at 4 h of erosive exposure. Values are average ± standard deviation, *n* = 4 for each group. A positive mean value indicates the erosion loss, a negative mean value indicates a mineral gain on the surface. The letters associated with each experimental group (A, B, or C) designate experimental groups that are statistically different at *p* < 0.05 (groups with the same letter are not statistically different) as determined by two-way ANOVA followed by post hoc ranked comparisons.

4 h	Fluoride (ug/g)											
Citric Acid (%)	0			25			50			100		
**0.00**	−16.0	±	5.7 A	−14.3	±	10.0 A	−9.5	±	5.1 A	−4.6	±	3.1 A
**0.25**	45.8	±	7.5 C	25.1	±	8.8 B	12.0	±	8.2 B	21.4	±	7.2 B
**1.00**	56.1	±	14.3 C	−5.8	±	8.3 A	12.0	±	12.3 B	9.9	±	4.7 B

**Table 3:** ANOVA with replication.

A. ANOVA (Two-Factor with Replication) 1 h					
*Source of Variation*	*SS*	*df*	*MS*	*F*	*p-Value*
Fluoride	7345.772	3	2448.591	53.25291	2.570 × 10^−13^
CA	3485.981	2	1742.991	37.90724	1.382 × 10^−9^
F × CA	6296.924	6	1049.487	22.82466	6.592 × 10^−11^
Within	1655.295	36	45.98041		
Total	18783.97	47			
**B. ANOVA (Two-Factor with Replication) 4 h**					
***Source of Variation***	***SS***	***df***	***MS***	***F***	***p-Value***
Fluoride	5269.387	3	1756.462	24.34624	8.800 × 10^−9^
CA	12238	2	6118.999	84.81514	2.387 × 10^−14^
F × CA	5941.714	6	990.2856	13.7263	4.782 × 10^−8^
Within	2597.225	36	72.14513		
Total	26046.32	47			
